# Particle Surface Modification in the Near-Electrode Region of an RF Discharge

**DOI:** 10.3390/nano11112931

**Published:** 2021-11-02

**Authors:** Evgenii Aleksandrovich Kononov, Mikhail Mikhailovich Vasiliev, Elena Valeryevna Vasilieva, Oleg Fedorovich Petrov

**Affiliations:** 1 Joint Institute for High Temperatures of the Russian Academy of Sciences, Izhorskaya st. 13 Bldg. 2, 125412 Moscow, Russia; mixxy@mail.ru (M.M.V.); elen_vasilieva@mail.ru (E.V.V.); ofpetrov@ihed.ras.ru (O.F.P.); 2 Moscow Institute of Physics and Technology, Institutskiy Pereulok 9, 141701 Dolgoprudny, Russia

**Keywords:** low-temperature plasma, surface etching, sputtering, plasma deposition, plasma modification

## Abstract

The results of a study on particles’ surfaces after being exposed to the near-electrode region of a radio frequency (RF) discharge are presented. It was experimentally displayed that metal starts being deposited on the surface of particles levitating above the lower electrode of the discharge chamber after switching the RF discharge on. For melamine-formaldehyde (MF) particles, the appearance of an island metal coating is observed after 30 min of plasma exposure. Eroded electrodes and elements of the gas discharge chamber may serve as a source of deposited material. In addition, an analysis of the surface and composition of particles placed on the upper electrode after 6 h of plasma exposure is presented. We reveal that the composition and structure of the particle coating changes during the experiment. The MF particles under exposure become eroded, and needle-like structures containing metals are formed on their surface. We also observe the formation of columnar structures from the products of erosion of electrodes on particles with a metal coating.

## 1. Introduction

RF discharges are widely used in various fields of science and technology [1], including studies of dusty plasma systems of charged particles [2]. In the plasma of an RF discharge, it is possible to form levitating Coulomb systems using micron-sized particles differing in shape and composition, as well as to observe a wide range of effects, resulting from various influences, such as changes in the parameters of a gas discharge [3]. Under external influences, such systems can exhibit active properties, as well as the ability to self-organize [4].

At the same time, it is known that the size and surface of the particles that form the Coulomb system can change in plasma [5,6]. The action of such an active medium as plasma can lead to the development of unique properties by macroparticles. As a result, their behavior in the Coulomb system can change dramatically: passive Brownian particles can become active, which may cause different phenomena, such as phase transitions, clustering, etc. [7]. There is also the problem of the formation of melting centers in crystal systems caused by moving particles, the source of which, presumably, are the electrodes. Thus, an extended study of the modification of particles in the near-electrode region of an RF discharge is of undoubted interest.

In this paper, a study of the composition and surface structure changes of polymer particles with a metal coating and without it, during their exposure to the plasma of an RF gas discharge is presented.

## 2. Materials and Methods

**Figure 1:**
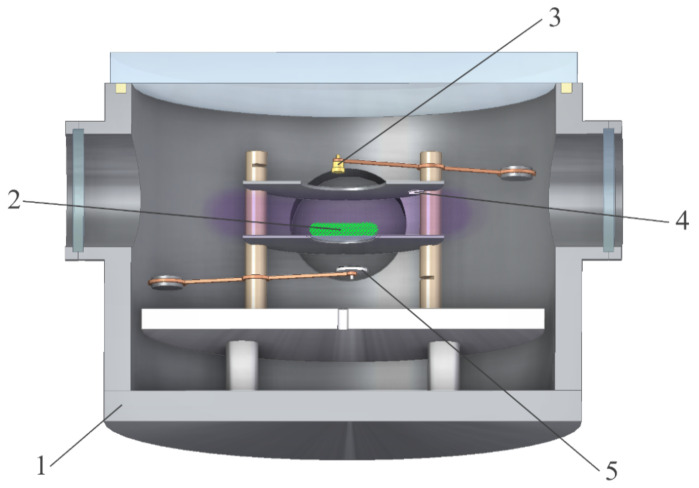
Experimental setup: 1—a discharge chamber; 2—a Coulomb system of particles above the grid; 3—a container with particles; 4—a draw-out unit with particles on the upper electrode; 5—a draw-out unit for collecting particles from the near-electrode layer.

Figure 1 shows a scheme of the experiment. The experiment is performed in the plasma of a capacitive radio frequency (RF) discharge generated between two disk-shaped electrodes within a gas discharge chamber. Electrodes with a diameter of 186 mm are located at a distance of 60 mm from each other. They are isolated and connected via an automatic matching device to an RF power supply by copper wires. A circular hole of 80 mm in diameter is made in the center of the upper steel electrode to be able to inject the particles into the discharge area and to record the processes occurring inside it. The injected particles move into the discharge and gain a negative charge because of ion and electron flows going to their surface; as a result, they start levitating in the near-electrode layer above the lower electrode due to the balance of gravity and electric forces. Additionally, there is a 71 mm diameter hole in the lower aluminum electrode, into which a steel grid with a mesh size of 2.4 mm is inserted. Such a configuration of the lower electrode is used to confine particles in a potential trap, so that the particles are trapped in the center of the near-electrode layer and form a Coulomb system. Under the lower electrode, there is a draw-out unit with a double-sided electrically conductive carbon tape, on which dust particles from the Coulomb system fall down after switching the discharge off. Additionally, we have collected particles, placed on another draw-out unit with carbon tape fixed on the upper electrode.

The gas discharge chamber is preliminary pumped out and filled with a plasma-forming gas, argon, up to a pressure of 5 Pa. During the experiment, the discharge chamber is continuously evacuated by a turbo-molecular pump, while the constant pressure is maintained by a continuous supply of the working gas at a rate of 2 standard cm^3^/min. In such conditions, the discharge plasma retains unchanged properties throughout the entire experiment. An alternating voltage is applied to the electrodes from a high-frequency generator with a frequency of 13.56 MHz, as a result of which a glow discharge appears. Plasma is generated at a power W_load_ = 15 W, while the reflected power is W_ref_ = 4 W.

Two types of monodisperse spherical melamine-formaldehyde (MF) particles, produced by MicroParticles GmbH (Berlin, Germany), are used in the experiments: with a diameter of 10.6 ± 0.1 μm without coating and with a diameter of 10.0 ± 0.2 μm with a copper coating (thickness of ≈200 nm). Each experiment is performed only with one type of particle, exposed to plasma in two different places: (1) uniform exposure of the entire surface while particles levitate in a discharge layer above the lower electrode (within the Coulomb system), (2) exposure of half of the surface while particles are laying on the upper electrode. After switching the discharge off, we take away particles from both places for further analysis.

A scanning electron microscope (SEM), a FEI Nova NanoSEM 650 (Thermo Fisher Scientific, Waltham, MA, USA), is used to analyze the original particles before plasma exposure and the extracted ones after exposure. The SEM method makes it possible to obtain an image of the surface of the material under study with a high spatial resolution (0.4 nm), as well as to carry out X-ray spectral microanalysis (EDAX Octane Pro, EDAX, Mahwah, NJ, USA) to obtain the elemental composition of the material under study.

## 3. Results

**Figure 2:**
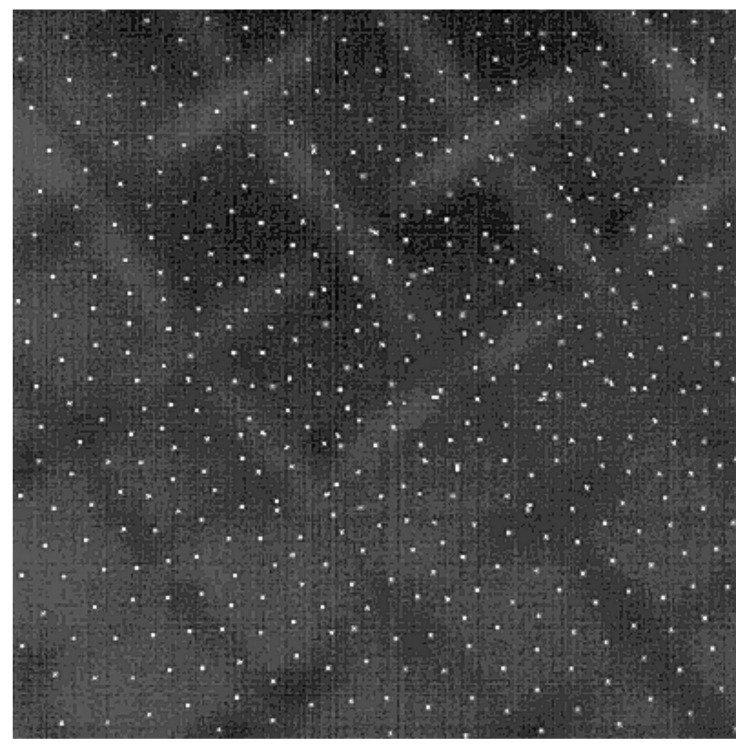
A frame of a video recording of the quasi-two-dimensional Coulomb system levitating above the grid.

**Figure 3:**
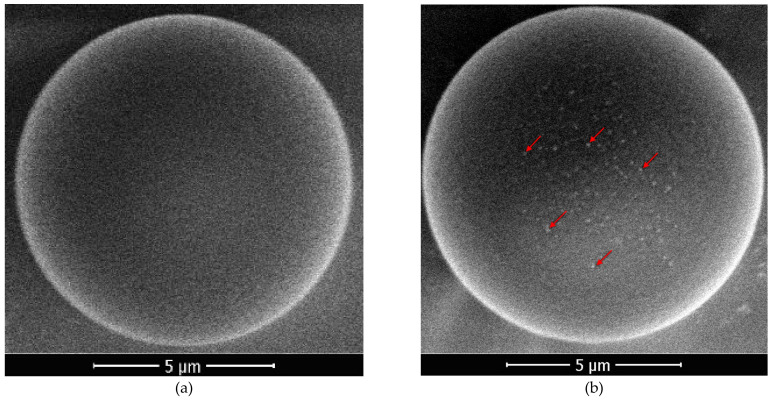
SEM images of the uncoated polymer particles (**a**) before and (**b**) after 30 min of plasma exposure in the Coulomb system. Red arrows mark island metal coating.

**Figure 4:**
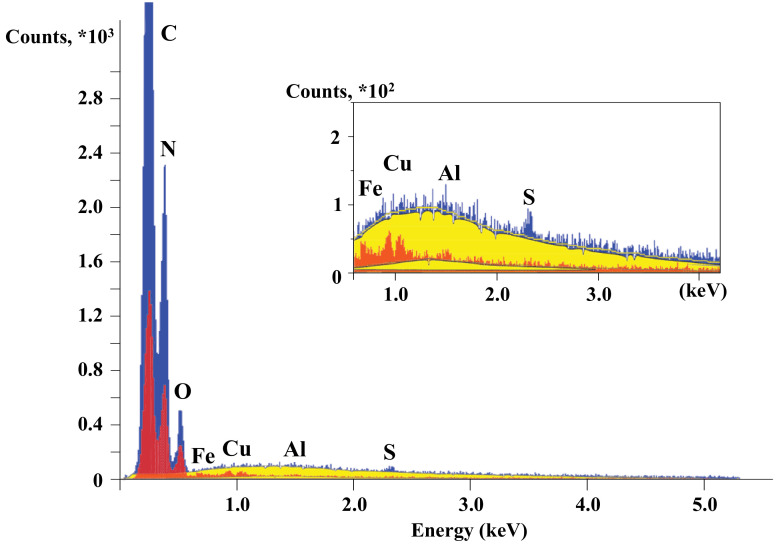
Spectra of the uncoated particles’ compositions before (blue) and after (red) 6 h of plasma exposure. Yellow is for the background.

**Figure 5:**
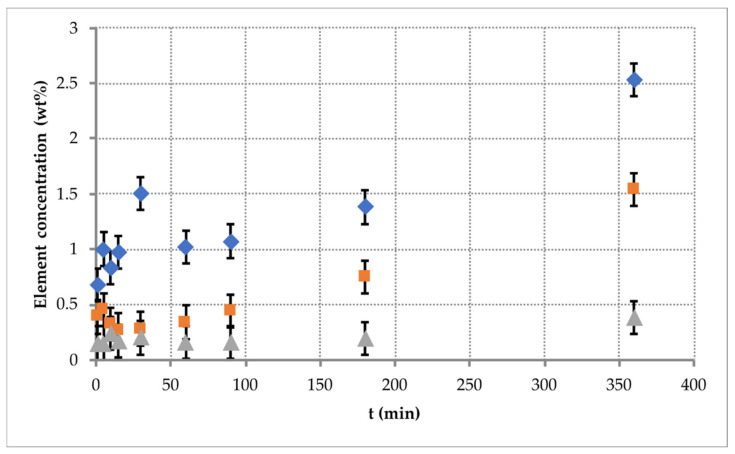
The element weight of various types of metal on the surface of MF particles levitating within the Coulomb system at different exposure times. Grey triangle is for Al, orange square is for Cu and blue rhomb is for Fe.

A major study is performed for MF particles without coating that form Coulomb systems above the lower electrode; the following results (see Table 1 and Figure 2, Figure 3, Figure 4, Figure 5) are presented for this set of particles. Figure 2 shows a typical picture of an obtained system, which is a quasi-two-dimensional system, consisting of ~2000 particles.

It is experimentally found that the composition and structure of the particles’ surfaces change: metals are deposited on their surface, and an island metal coating appears (see Figure 3) after 30 min of exposure or more. The size of the islands is 100–200 nm after 30 min of exposure, and the centers of crystallization are distributed non-uniformly over the surface of the particles.

The surface composition of the original particles before exposure as well as the particles after exposure that have fallen down on the carbon tape of the draw-out unit below the grid after switching the discharge off has been also analyzed. The results of X-ray spectral microanalysis are presented in Figure 4 and Figure 5 as well as in Table 1. The spectra (see Figure 4) show, for captured particles, the appearance of metal peaks, the sources of which can be steel and aluminum electrodes, as well as copper wires, which connect the electrodes with the RF generator. When exposed to low-energy (Ei ~ 100 eV) ion flows [8], the erosion of electrodes and other metal surfaces in contact with the plasma occurs. Erosion products are deposited on the surface of the particles and form an island coating. The metal content varies among particles and reaches 2.53 wt% for iron, 1.54 wt% for copper and 0.38 wt% for aluminum after 6 h of exposure to plasma.

Looking at the time dependence of the amount of metal on the surface of the particles (Figure 5 and Table 1), one can see that the metal starts being deposited on the surface of the particles after only 1 min of exposure, and its amount increases over time. At the same time, the change in the elemental composition of the surface is nonlinear in time. In addition, the island coating is not observed before times of up to 30 min of exposure.

Contrastingly, no changes in the surface structure of particles with a copper coating, levitating in Coulomb system, have been observed over the whole duration of the experiment (6 h), although the elemental composition of the surface has changed (see Table 2).

**Figure 6:**
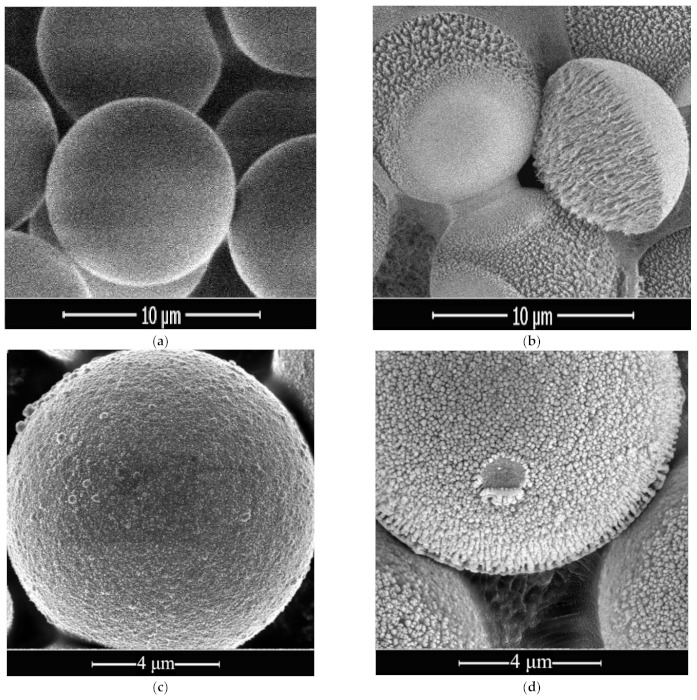
SEM images of the MF particles without coating (**upper row**) and with a copper coating (**lower row**): (**a**,**c**)—original particles before plasma exposure; (**b**,**d**)—particles after 6 h of plasma exposure laying on the upper electrode.

We also analyzed the composition and surface structure of both types of particles, laying on the upper electrode, after 6 h of plasma exposure (see Figure 6). Polymer particles without coating obtain an ellipsoidal shape due to erosion (see Figure 6b). At the same time, metal is deposited on the half of their surface facing the plasma, resulting in the formation of needle-like structures. The needles have the following typical dimensions: a length of 1–1.5 μm and a thickness of 10–300 nm. As for the particles with a copper coating, laying on the upper electrode, we observed the formation of column-like structures on their surface (see Figure 6d). The typical column has a length of 100–200 nm and a thickness of 50–100 nm.

Finally, we present the elemental composition of the surface of the original particles before and after 6 h of plasma exposure, collected from the upper electrode and the Coulomb system. Mass analysis shows that the columnar formations on the surface of the exposed particles (Figure 6b,d) consist mainly of iron (up to 3.86 wt%, Table 2). At the same time, particles with a copper coating collected from the Coulomb system contain more external inclusions after plasma exposure than those collected from the upper electrode. We should note that the particles on the upper electrode do not always form a single layer, as a result of which a different degree of surface modification is obtained due to partial screening by the overlying particles.

To sum up, particles in the near-electrode region of the discharge are modified due to the erosion of electrodes and other metal surfaces, which are in contact with the discharge plasma. Moreover, such a modification leads to the development of unique functional properties of the surface and composition by the particles, which can be used both for medical [9] and technical [10] purposes (targeted drug delivery and the creation of powder bases for composite materials), as well as scientific ones [11] (creation of active Janus particles for studying self-organization in colloidal systems).

## 4. Discussion

Plasma containing microparticles is widespread in nature (especially in space), in technological processes, etc. [12]. Particles in plasma significantly affect its properties, for example, the distribution of charges, fields, etc. In turn, the plasma interacts with objects which are in contact with it, such as different parts of the experimental setup (electrodes or walls of the working chamber) as well as dust particles, causing a change in the properties of the latter. It is important to take this fact into account when describing dusty plasma systems, their properties and the processes taking place in such systems.

For example, in theoretical works [13,14], transport processes and phase transitions in a system of spherical polymer particles are described; in experimental research [3], phase transitions in a system of such particles are investigated when the plasma parameters are changed. Another example is shown in [15], where the presence of a metal cover on particles leads to phase transitions in the Coulomb system because of the external action of a laser under the constant parameters of the gas discharge. On the other hand, research [7] shows that a phase transition is also possible in a Coulomb system of uncoated particles as a result of scattering laser radiation by particles under constant plasma parameters. Polymer particles become modified and acquire a radiation-absorbing metal coating, which, under laser action, leads to heating of macro-particles and the appearance of a photophoretic force contributing to their motion.

In this work, we present convincing evidence that particles become modified in the near-electrode region of an RF discharge, as a result of which the properties of their surfaces change. Additionally, this fact should be taken into account in further research and applications of particle systems in plasma.

## 5. Conclusions

In this work, we have studied the composition and surface structure changes of polymer particles with and without a metal coating during their exposure to two regimes: while levitating in the near-electrode region of an RF discharge, and laying on the upper electrode. When the particles levitate above the grid forming the Coulomb system, different types of metal are deposited on their surface; at this point, the change in the elemental composition of the surface is nonlinear in time. On the surface of uncoated particles, a metallic island film starts forming, which probably may evolve into a continuous coating over time. The source of the material is electrodes and other metal surfaces exposed to sputtering by low-energy (E ~ 100 eV) plasma-forming gas ions.

As for particles collected from the upper electrode, uncoated particles erode, as a result of which acicular structures of deposited metals can form on the surface facing the plasma, and the shape of the macroparticles can change. At the same time, columnar structures consisting of the products of electrode erosion are formed on the surface of copper-coated particles.

## Figures and Tables

**Table 1:** Elemental composition of polymer particles, without coating, levitating within the Coulomb system depending on the exposure time.

	Element (wt%)						
Time (min)	Fe	Cu	Al	C	N	O	S
0				31.85	55.8	11.98	0.37
1	0.68	0.39	0.15	37.02	52.75	8.65	0.36
5	1	0.45	0.15	38.8	49.76	9.5	0.34
10	0.83	0.32	0.24	38.7	50.19	9.38	0.34
15	0.97	0.27	0.17	38.78	49.86	9.58	0.37
30	1.5	0.28	0.2	38.51	49.63	9.49	0.39
60	1.02	0.34	0.16	37.62	51.76	8.77	0.33
90	1.07	0.44	0.16	37.3	52.03	8.64	0.36
180	1.38	0.75	0.19	35.99	51.77	9.63	0.29
360	2.53	1.54	0.38	37.71	45.94	11.53	0.37

**Table 2:** Elemental composition of the MF particles before and after 6 h of exposure, collected from the upper electrode and from the Coulomb system.

	Element (wt%)							
Particles	Fe	Cu	Al	C	N	O	S	Cl
**Without coating**								
Before plasma exposure				31.85	55.8	11.98	0.37	
Exposed in the Coulomb system	2.53	1.54	0.38	37.71	45.94	11.53	0.37	
Exposed on the upper electrode	2.55	0.38	0.29	32.31	51.14	12.93	0.4	
**With Cu coating**								
Before plasma exposure		84.68		4.45	6.95	2.56		1.36
Exposed in the Coulomb system	4.59	71.87	0.47	7.21	10.82	3.59		1.45
Exposed on the upper electrode	3.86	53.78	0.35	12.29	21.58	5.52	0.66	1.96

## Data Availability

The data is available on reasonable request from the corresponding author.
